# Different Arbuscular Mycorrhizal Fungi Cocolonizing on a Single Plant Root System Recruit Distinct Microbiomes

**DOI:** 10.1128/mSystems.00929-20

**Published:** 2020-12-15

**Authors:** Jiachao Zhou, Xiaofen Chai, Lin Zhang, Timothy S. George, Fei Wang, Gu Feng

**Affiliations:** a College of Resources and Environmental Sciences, China Agricultural University, Beijing, China; b The James Hutton Institute, Invergowrie, Dundee, United Kingdom; c School of Resource and Environmental Sciences, Henan Institute of Science and Technology, Xinxiang, China; Institute of Soil Science, Chinese Academy of Sciences

**Keywords:** ^13^C-DNA-SIP, arbuscular mycorrhizae, COG, hyphal exudates, hyphosphere, microbiome, mycorrhizal pathway

## Abstract

Plant roots are usually colonized by various arbuscular mycorrhizal (AM) fungal species, which vary in morphological, physiological, and genetic traits. This colonization constitutes the mycorrhizal nutrient uptake pathway (MP) and supplements the pathway through roots. Simultaneously, the extraradical hyphae of each AM fungus is associated with a community of bacteria. However, whether the community structure and function of the microbiome on the extraradical hyphae differ between AM fungal species remains unknown. In order to understand the community structure and the predicted functions of the microbiome associated with different AM fungal species, a split-root compartmented rhizobox cultivation system, which allowed us to inoculate two AM fungal species separately in two root compartments, was used. We inoculated two separate AM fungal species combinations, (i) Funneliformis mosseae and Gigaspora margarita and (ii) Rhizophagus intraradices and *G. margarita*, on a single root system of cotton. The hyphal exudate-fed, active microbiome was measured by combining ^13^C-DNA stable isotope probing with MiSeq sequencing. We found that different AM fungal species, which were simultaneously colonizing a single root system, hosted active microbiomes that were distinct from one another. Moreover, the predicted potential functions of the different microbiomes were distinct. We conclude that the arbuscular mycorrhizal fungal component of the system is responsible for the recruitment of distinct microbiomes in the hyphosphere. The potential significance of the predicted functions of the microbial ecosystem services is discussed.

**IMPORTANCE** Arbuscular mycorrhizal (AM) fungi form tight symbiotic relationships with the majority of terrestrial plants and play critical roles in plant P acquisition, adding a further dimension of complexity. The plant-AM fungus-bacterium system is considered a continuum, with the bacteria colonizing not only the plant roots, but also the associated mycorrhizal hyphal network, known as the hyphosphere microbiome. Plant roots are usually colonized by different AM fungal species which form an independent phosphorus uptake pathway from the root pathway, i.e., the mycorrhizal pathway. The community structure and function of the hyphosphere microbiome of different AM species are completely unknown. In this novel study, we found that arbuscular mycorrhizal fungi cocolonizing on single plant roots recruit their own specific microbiomes, which should be considered in evaluating plant microbiome form and function. Our findings demonstrate the importance of understanding trophic interactions in order to gain insight into the plant-AM fungus-bacterium symbiosis.

## Abstract (precis)

Arbuscular mycorrhizal (AM) fungi form tight symbiotic relationships with the majority of terrestrial plants and play critical roles in plant P acquisition, adding a further dimension of complexity. The plant-AM fungus-bacterium system is considered a continuum, with the bacteria colonizing not only the plant roots, but also the associated mycorrhizal hyphal network, known as the hyphosphere microbiome. Plant roots are usually colonized by different AM fungal species which form an independent phosphorus uptake pathway from the root pathway, i.e., the mycorrhizal pathway.

## INTRODUCTION

Plant-arbuscular mycorrhizal (AM) fungal symbiosis has existed for over 460 million years (1). Consequently, over 80% of terrestrial plants form a symbiosis with arbuscular mycorrhizal (AM) fungi for efficient nutrient uptake or to confer resistance to stress (2). Exploitation of these symbioses is of high environmental and economic value (3). Like plant roots, AM fungi produce large networks of extraradical hyphae in the soil, release carbon, and recruit free-living soil microbes to colonize the hyphae (4–8). In recent years, an intimate cooperative relationship between AM fungal hyphae and bacteria has been observed, supported by multiple lines of evidence, including both microscopic observations (9) and molecular analyses (5). Bacteria associated with AM fungi (hyphosphere) have been identified as the third component of plant-AM fungal symbiosis because of the critical role they play in mycorrhizal function (3, 6, 10, 11). Revealing the secrets of hyphosphere microbiomes is essential for a better understanding of the belowground ecosystem.

Many soil factors, such as pH and spatial structure, have been identified to influence the bacterial community associated with plant roots, while AM fungi were also identified as a major determining factor (12). In natural and agricultural systems, the root system of a mycorrhizal plant is usually simultaneously colonized by diverse AM fungal species (13). The cocolonizing AM fungi have different morphological, physiological, and genetic characteristics (14–19). The coexisting AM fungal species show different contributions to the growth and P uptake of the host plant (16). For example, Glomus intraradices can rapidly colonize available P patches beyond the root surface and transport significant amounts of P toward the roots, while Glomus margarita has been shown to provide P benefits to the plants by forming dense mycelium networks close to the roots where remaining soil P was less available (16). In addition, recent decoding of the whole-genome sequences of AM fungi suggest that there is large variation in the genetic control of functions (17), e.g., Glomus rosea contains a much larger secretome size and more secreted proteins (SSP) than *Rhizophagus* spp. (17). Collectively, the above-described morphological, physiological, and genetic differences indicate that the hyphal exudates of AM fungal species are likely to be different, which in turn, is likely to lead to differences in the hyphosphere microbial community structure and function. However, at present, no direct evidence exists that shows the difference between fungal species cocolonizing on a single plant root system. Therefore, to uncover such a difference is fundamental for understanding the central question in fungus-bacterium interaction research: how bacteria and mycorrhizal fungi associate and become mutually beneficial neighbors (3).

Several factors may affect the results of hyphosphere microbial community composition in the plant-AM fungi-soil system. First, plant root exudates are an important factor in the recruitment of the soil microbial community. In order to get direct evidence of the effect of hyphal exudates on hyphosphere microbiome characteristics, it is essential to separate their influence from that of the root exudates. Second, the vitality of AM fungal hyphae is important. Previous studies have shown that soil bacteria differ in their ability to colonize vital and nonvital hyphae and that this can also be influenced by the arbuscular mycorrhizal fungal species involved (20). Therefore, a method that can test the vital and nonvital hyphae is necessary to identify the hyphosphere microbiome. Third, the feedback effects of plants on the growth of AM fungi due to changes in plant physiology induced by the fungi (21, 22) are critical. In the past, split-root methods were used to quantify C allocation to different AM fungal species cocolonizing on a single root system of a plant (23) in order to assess this factor.

In this study, we hypothesized that the different AM fungal species that colonized on a single root system would recruit distinct microbiomes. To test our hypothesis, we developed a new integrated approach where we grew cotton (Gossypium hirsutum L.) plants in a split-root and compartmented rhizobox in which a buffer zone was set to prevent root exudates from diffusing into the hyphal compartment and to avoid feedback effects. Two independent experiments (experiment 1 [Exp 2] and experiment 2 [Exp 2]) were performed. In Exp 1, we inoculated two different AM fungal species, Funneliformis mossea and Gigaspora margarita, to two separate root compartments, while in Exp 2, Rhizophagus intraradices and *Gigaspora margarita* were inoculated to the two root compartments. We used ^13^CO_2_ to pulse-label the plant-AM fungus-hypha-associated bacteria during the last week before harvest and tested active hypha-associated microbiomes by ^13^C-DNA-SIP (stable isotopic probing) methods and MiSeq high-throughput sequencing.

## RESULTS

### Mycorrhizal colonization.

**FIG 1:**
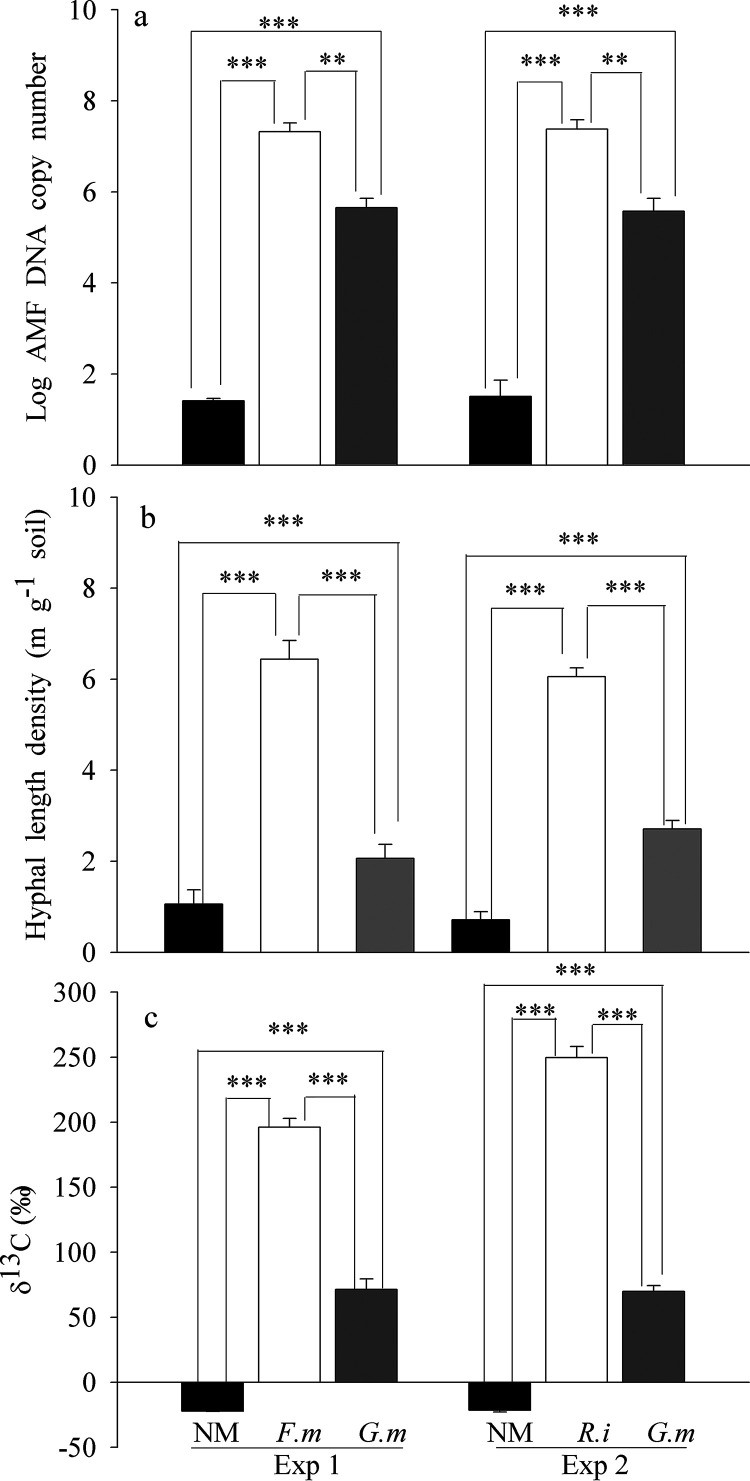
(a to c) The log AM fungal DNA copy number in the roots of cotton plants (a), hyphal length density (b), and ^13^C abundance of the HC soil (c). Exp 1 and Exp 2 refer to the two independent experiments. The nonmycorrhizal (NM) control is compared to *Rhizophagus intraradices* (*R.i*) (EY108), *Funneliformis mosseae* (*F.m*) (MD118), and *Gigaspora margarita* (*G.m*) (JA101A), the three different AM fungal inocula. All the treatments shown in this part were ^13^C labeled. **, *P* < 0.01; ***, *P* < 0.001.

We used the DNA copy number in roots to indicate the colonization of each AM fungal species. Based on the principles of quantitative PCR (qPCR), any measurement that is less than 100 copies can be considered background and indicative of a lack of presence of mycorrhizal DNA (19). In nonmycorrhizal (NM) controls in both Exp 1 and Exp 2, the root AM fungal DNA copy number was below this threshold, indicating that no AM fungus was detected in the roots. Both species of AM fungi were able to colonize the root system of the same plant effectively at the same time. In Exp 1, after inoculation with *F. mosseae*, the root AM fungal DNA copy number significantly increased to 10^7^, and inoculation with *G. margarita* increased the AM fungal DNA copy number to 10^5^, which was significantly less than that of the concomitant inoculation with *F. mosseae* (*P < *0.01) (Fig. 1a). In Exp 2, after inoculation with *R. intraradices*, the root AM fungal DNA copy number significantly increased to 10^7^, and inoculation with *G. margarita* increased the AM fungal DNA copy number to 10^5^, significantly less than that of concomitant inoculation with *R. intraradices* (*P < *0.01) (Fig. 1a).

### Hyphal length density in hyphal compartment (HC) soil.

In the NM control in both Exp 1 and Exp 2, less than 0.6 m g^−1^ soil of hyphae was detected, implying that there were some saprotrophic fungi in the compartments. In both experiments, the hyphal length density of *F. mosseae* and *R. intraradices* was more than 6 m g^−1^ soil, while the density of *G. margarita* was about 3 m g^−1^ soil. *G. margarita* produced significantly (*P < *0.001) less hyphal length than *F. mosseae* and *R. intraradices* in both experiments (Fig. 1b).

### Biomass, P concentration, and P content of shoot.

The cotton plants grew well after being transplanted into the split-root microcosm. At harvest, the shoot biomass and P concentration and P content data of all inoculation treatments were significantly (*P < *0.01) greater than those of their corresponding NM control treatments (Table 1).

**TABLE 1:** Biomass, phosphorus (P) concentration, and P content of shoots in different inoculation treatments^*a*^

Conditions	Biomass (g)	P concn (mg g^−1^)	P content (mg plant^−1^)
Exp 1			
NM	0.82 ± 0.06	0.91 ± 0.04	0.74 ± 0.05
* F.m*/*G.m*	5.93 ± 0.25**	1.49 ± 0.06*	8.84 ± 0.47***
Exp 2			
NM	0.79 ± 0.06	0.87 ± 0.07	0.67 ± 0.04
*R.i*/*G.m*	5.78 ± 0.30**	1.92 ± 0.09**	11.03 ± 0.59***

a Exp 1 and Exp 2 refer to two independent experiments. The nonmycorrhizal (NM) control is compared to *F.m/G.m or R.i/G.m* inoculation treatments in two independent experiments. All the treatments shown in this part were ^13^C labeled. The values in the table were the mean value. *, *P* < 0.05; **, *P* < 0.01; ***, *P* < 0.001.

### ^13^C incorporation of HCs soil and bacteria.

The DNA of targeted bacterial populations in the hyphosphere was successfully labeled with ^13^C. In the NM control, the isotopic signature (δ^13^C) of hyphosphere soil was consistent with the atmospheric concentration (approximately –20‰). The isotopic signatures in the HCs of inoculated treatments were greater than that of the NM control (Fig. 1c). In addition, inoculation with *F. mosseae* and *R*. *intraradices* resulted in much greater ^13^C abundance than that of *G. margarita* in Exp 1 and Exp 2, respectively (Fig. 1c). The incorporation of ^13^C into bacterial DNA in the hyphosphere soil was corroborated by parallel incubation of microcosms labeled with ^12^C. The gradients in all ^12^C-labeled soil after 7 days clearly showed peaks of bacterial DNA in a light DNA fraction. In contrast, the bacterial DNA in all ^13^C-labeled soil had apparently shifted toward heavier buoyant densities (Fig. S1).

10.1128/mSystems.00929-20.3 FIG S1 Quantitative distribution of density-resolved bacterial 16S rDNA obtained from hyphospheres of different inoculation treatments after a 7-day labeling period with ^13^CO_2_ and ^12^CO_2_. Bacterial template distribution within DNA gradient fractions was quantified with real-time qPCR. The normalized data are the ratio of the copy number in each gradient fraction to the maximum quantities from each treatment. The DNA fractions subjected to pyrosequencing analysis are marked with arrows. Download FIG S1, TIF file, 0.1 MB. Copyright © 2020 Zhou et al. 2020 Zhou et al. This content is distributed under the terms of the Creative Commons Attribution 4.0 International license.

### Taxonomic profiling of bacteria associated with AM fungal hyphae.

The DNA from the selected fractions shown in Fig. S1 was sequenced using a high-throughput MiSeq PE 300 platform. After quality filtering and standardizing of the raw data, a data set of 1,989,255 high-quality sequences with an average length of 439 bp and over 24,433 reads per sample was generated. At 97% similarity, the number of operational taxonomic units (OTUs) ranged from 505 to 836, depending on the sample. The microbiome of ^13^C-labeled samples was considered the active one, which was influenced by the hyphae directly (24). Thus, the following analyses were all based on the ^13^C-labeled active samples.

### The effect of AM fungi hyphae on the soil microbiome.

The NM control contained a higher species richness and Shannon index but a lower Simpson index than the inoculated treatments (Table 2). After aligning the OTUs with the Greengenes database, the soil microbial community was classified into phylotypes consisting of 10 dominant phyla and others. The dominant taxa included *Proteobacteria*, *Actinobacteria*, *Firmicutes* and *Gemmatimonadetes*, *Bacteroidetes*, *Chloroflexi*, *Acidobacteria*, *Cyanobacteria*, *Planctomycetes*, and *Fusobacteria*, which contributed to over 95% of the whole community under all conditions (Fig. S2). There was a significant difference in the abundance of some taxa compared with the NM control after inoculation. However, the difference in taxon abundance was dependent on the AM fungal species (Fig. S2). For example, compared to the NM control, (i) the hyphosphere of *F. mosseae* contained a greater abundance of *Actinobacteria* and *Gemmatimonadetes* but contained fewer *Proteobacteria*, *Bacteroidetes*, *Acidobacteria*, and *Planctomycetes*; (ii) the hyphosphere of *R. intraradices* contained a greater abundance of *Actinobacteria* and *Firmicutes* but contained fewer *Proteobacteria* and *Bacteroidetes*; and (iii) the hyphosphere of *G. margarita* contained a greater abundance of *Proteobacteria*, *Cyanobacteria*, and *Fusobacteria* but fewer *Gemmatimonadetes*, *Chloroflexi*, *Acidobacteria*, and *Planctomycetes* (Fig. S2). In addition, the principal-component analysis (PCA) also showed that the community structure of the inoculated hyphal compartments was different from that of the NM control (Fig. S3).

**TABLE 2:** α-diversity indexes in different inoculation treatments^*a*^

Conditions	Species richness	Shannon diversity	Simpson diversity
Exp 1			
NM	818 ± 62 a	4.80 ± 0.08 a	0.024 ± 0.003 b
*F.m*	700 ± 64 b	4.15 ± 0.14 c	0.051 ± 0.010 a
*G.m*	659 ± 82 b	4.57 ± 0.40 b	0.047 ± 0.021 a
Exp 2			
NM	816 ± 100 a	4.85 ± 0.08 a	0.022 ± 0.002 c
*R.i*	659 ± 30 b	4.51 ± 0.08 b	0.034 ± 0.002 b
*G.m*	539 ± 99 c	4.47 ± 0.40 b	0.048 ± 0.014 a

a Exp 1 and Exp 2 refer to two independent experiments. The nonmycorrhizal (NM) control is compared to *Rhizophagus intraradices* (*R.i*) (EY108), *Funneliformis mosseae* (*F.m*) (MD118), and *Gigaspora margarita* (*G.m*) (JA101A), the three different AM fungal inocula. All the treatments shown in this part were ^13^C labeled. The values in the table were the mean value, and the different lowercase letters (a, b, and c) mean significance in the *P* < 0.05 level.

10.1128/mSystems.00929-20.4 FIG S2 Taxonomic assignment of sequence data at the phylum level. “Others” includes low-abundance taxa (<1% of the population). All relative abundance data were the mean of three samples. Exp 1 and Exp 2 refer to two independent experiments. The nonmycorrhizal (NM) control is compared to *Rhizophagus intraradices* (*R.i*) (EY108), *Funneliformis mosseae* (*F.m*) (MD118), and *Gigaspora margarita* (*G.m*) (JA101A), the three different AM fungal inocula. All samples are shown in this part. Download FIG S2, TIF file, 0.1 MB. Copyright © 2020 Zhou et al. 2020 Zhou et al. This content is distributed under the terms of the Creative Commons Attribution 4.0 International license.

### The difference between microbial diversity associated with the hyphae of different AM fungi.

**FIG 2:**
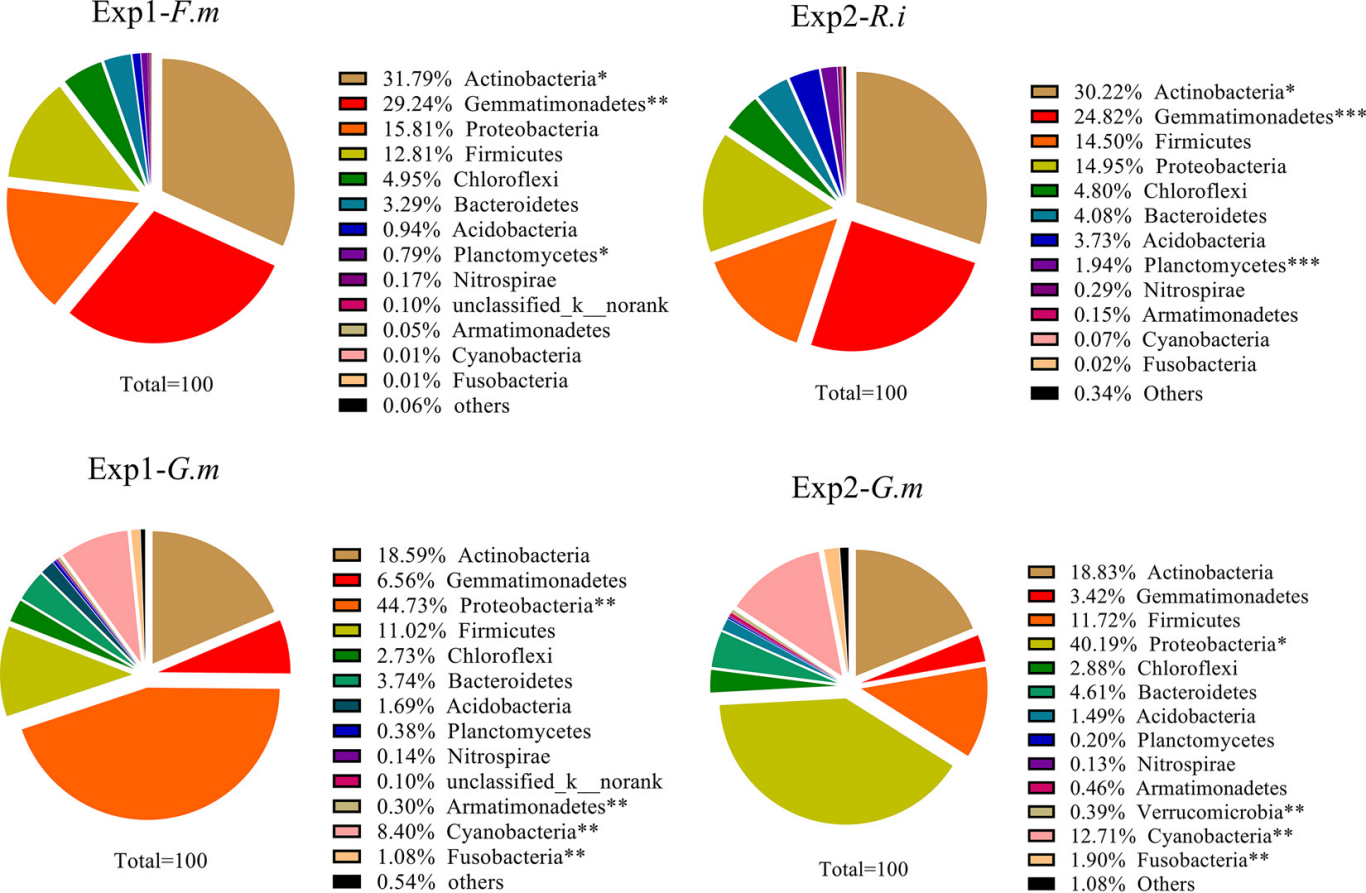
Phylum level distribution of DNA sequences. Exp 1 and Exp 2 refer to two independent experiments. The three different AM fungal inocula were *Rhizophagus intraradices* (*R.i*) (EY108), *Funneliformis mosseae* (*F.m*) (MD118), and *Gigaspora margarita* (*G.m*) (JA101A). All the treatments shown were ^13^C labeled. *, **, and ** mean this phylum was in greater abundance under this condition in same experiment in the *P < *0.05, 0.01, or 0.001 level, respectively.

**FIG 3:**
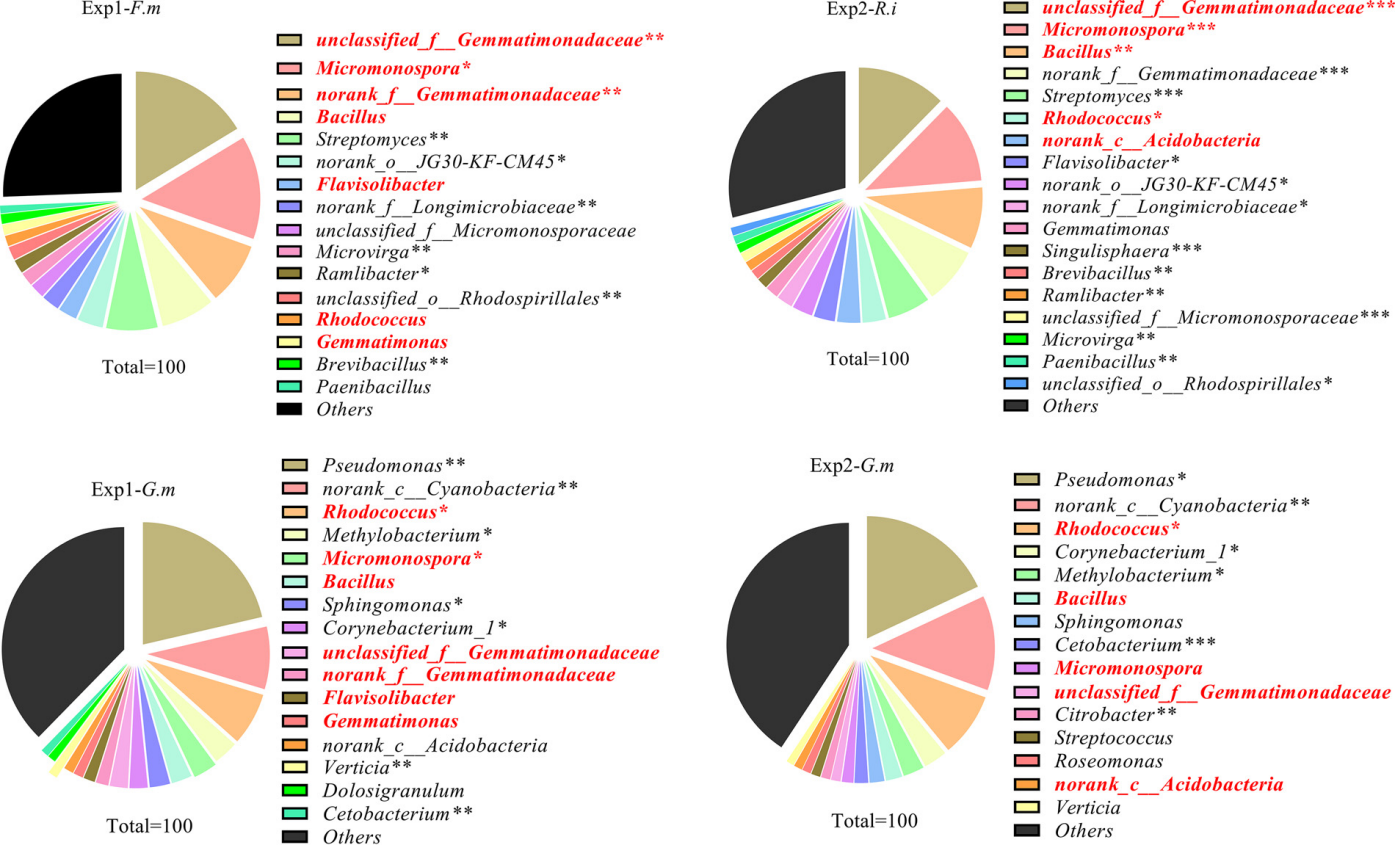
Genus-level distribution of DNA sequences. The genera showed in this plot were dominant, occupying over 1%, while other genera are summed in “Others.” Exp 1 and Exp 2 refer to two independent experiments. The three different AM fungal inocula were *Rhizophagus intraradices* (*R.i*) (EY108), *Funneliformis mosseae* (*F.m*) (MD118), and *Gigaspora margarita* (*G.m*) (JA101A). All the treatments shown in this part were ^13^C labeled. *, **, and ** mean this genus was in greater abundance under this condition in same experiment in the *P < *0.05, 0.01, or 0.001 level, respectively. The genera in red type occurred in both AM fungal hyphospheres of same experiment.

In Exp 1, there was no difference observed in number of OTUs in the *F. mosseae* hyphosphere microbiome compared to that of *G. margarita*, while in Exp 2, 100 more OTUs were observed in the *R. intraradices* hyphosphere microbiome than that of *G. margarita* (Table 2). In addition, there was a significant difference in the abundance of different taxa between different AM fungal species (Fig. 2 and 3). The species richness showed a similar result (Table 2). However, the *F. mosseae* hyphosphere exhibited a higher Shannon index than that of *G. margarita*, while the *R. intraradices* hyphosphere exhibited a higher Simpson index than that of *G. margarita* (Table 2). At the phylum level, the abundance of *Proteobacteria*, *Cyanobacteria*, and *Fusobacteria* in the hyphosphere of *G. margarita* was much greater than that of *F. mosseae* and *R. intraradices* in both Exp 1 and Exp 2. However, *G. margarita* exhibited a lower abundance of *Actinobacteria*, *Gemmatimonadetes*, and *Planctomycetes* (Fig. 2). There was no significant difference in the abundance of *Firmicutes*, *Chloroflexi*, *Bacteroidetes*, and *Acidobacteria* between *F. mosseae* and *G. margarita* or between *R. intraradices* and *G. margarita* in Exp 1 and Exp 2, respectively (Fig. 2).

**FIG 4:**
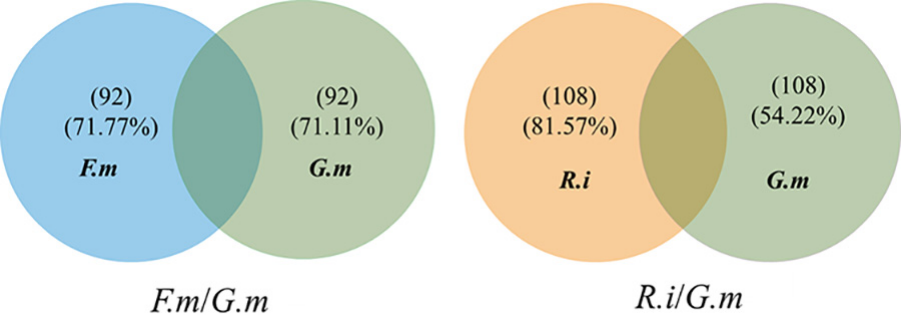
Venn plot of the number and proportion of genera in *Funneliformis mosseae* (*F.m*)/*Gigaspora margarita* (*G.m*) of Exp 1 and *Rhizophagus intraradices* (*R.i*)/*G.m* of Exp 2. The overlapping area refers to the genera without a significant difference in relative abundance among different inoculation treatments (^13^C samples), while the numbers and percentages in parentheses represent the percentage of genera with a significant difference in relative abundance among different inoculation treatments (^13^C samples). The three different AM fungal inocula were *Rhizophagus intraradices* (*R.i*) (EY108), *Funneliformis mosseae* (*F.m*) (MD118), and *Gigaspora margarita* (*G.m*) (JA101A). All the treatments shown in this part were ^13^C labeled.

**FIG 5:**
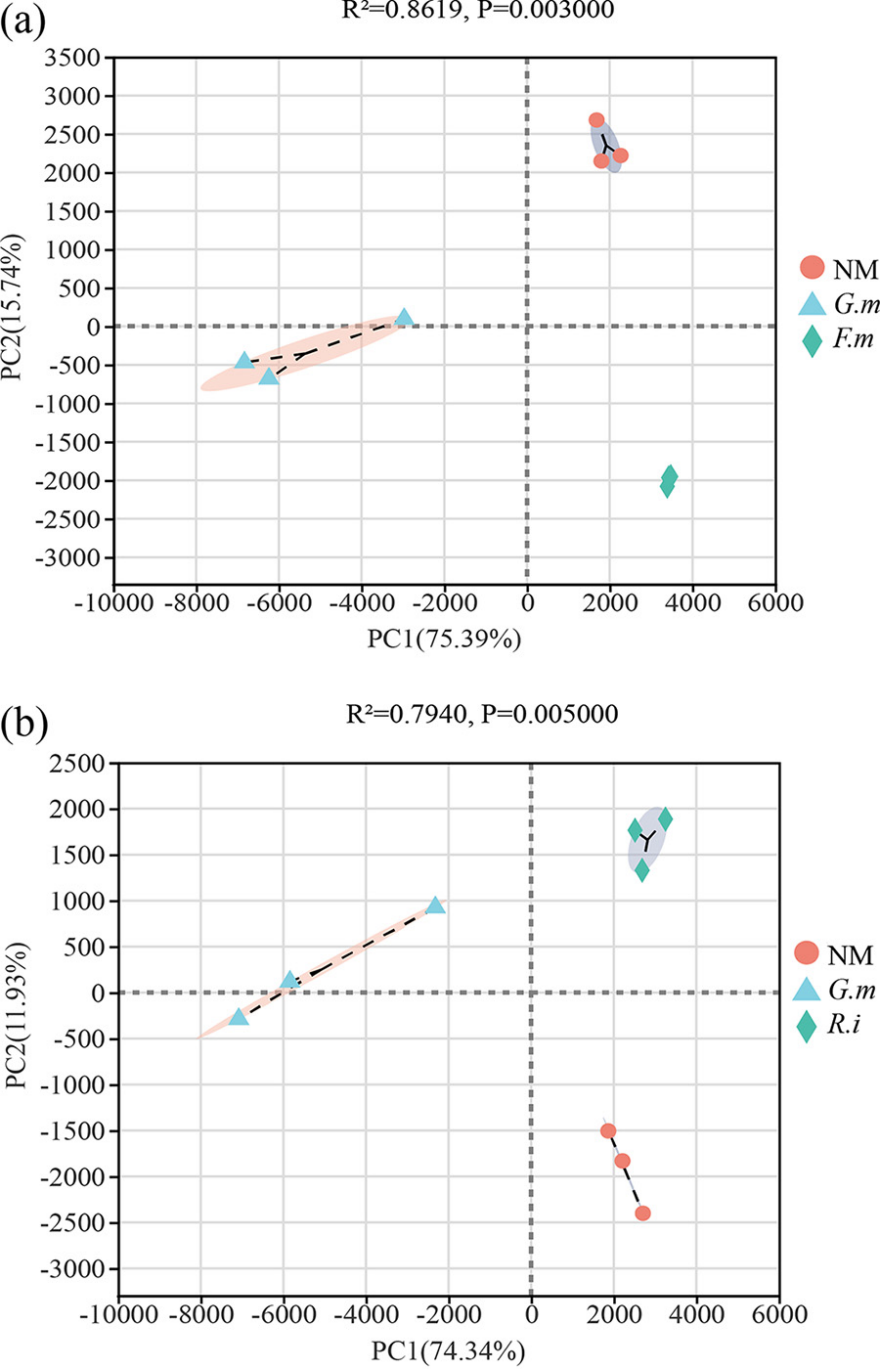
The principal-component analysis (PCA) of 16S rRNA genes in Exp 1 (a) and Exp 2 (b). Exp 1 and Exp 2 refer to two independent experiments. The nonmycorrhizal (NM) control is compared to *Rhizophagus intraradices* (*R.i*) (EY108), *Funneliformis mosseae* (*F.m*) (MD118), and *Gigaspora margarita* (*G.m*) (JA101A), the three different AM fungal inocula. The *P* value in this part was analyzed by permutational MANOVA (e.g., Adonis).

At the genus level, a total of 733 genera were observed in this study. We only considered the genus whose abundance was over 1% as the dominant taxon. In Exp1, 16 genera were identified as dominant taxa in both the *F. mosseae* and *G. margarita* hyphosphere microbiomes. However, only 7 of these were dominant in two different AM fungal hyphospheres. In Exp 2, 18 genera and 15 genera were identified as dominant in the *R. intraradices* and *G. margarita* hyphospheres, respectively. Only 5 genera were dominant in these two different AM fungal hyphospheres in Exp 2. Of all the genera, 92, which contained most of the dominant genera, were observed as being different between the microbiomes associated with *F. mosseae* and *G. margarita* extraradical hyphae in Exp 1; this represented approximately 70% of the total abundance. Likewise, 108 genera, which contained most of the dominant genera, were observed as being different between *G. margarita* and *R. intraradices* in Exp 2, representing over 80% of the total abundance in the *R. intraradices* hyphosphere and over 50% in the *G. margarita* hyphosphere (Fig. 3 and 4). In accordance with this, the PCA demonstrated that there was a significant difference between *F. mosseae* and *G. margarita* or *R. intraradices* and *G. margarita* community structure in Exp 1 and Exp 2, respectively (Fig. 5).

### The Cluster of Ortholog Genes (COG) functional pathway prediction.

**FIG 6:**
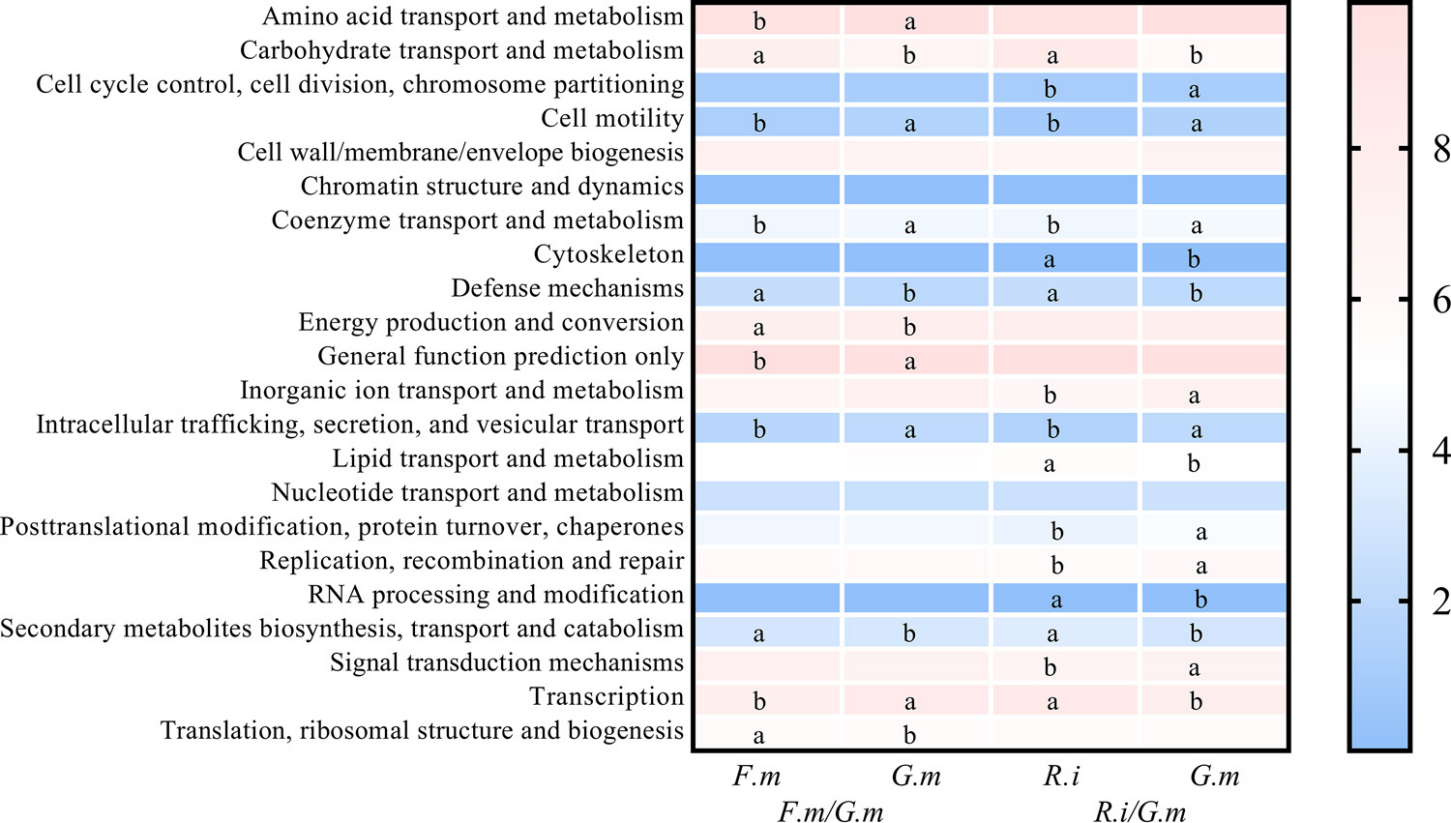
Heatmap plot of Cluster of Ortholog Genes (COG) functional pathway relative abundance in *Funneliformis mosseae* (*F.m*)/*Gigaspora margarita* (*G.m*) of Exp 1 and *Rhizophagus intraradices* (*R.i*)/*G.m* of Exp 2. The three different AM fungal inocula were *Rhizophagus intraradices* (*R.i*) (EY108), *Funneliformis mosseae* (*F.m*) (MD118), and *Gigaspora margarita* (*G.m*) (JA101A). All the treatments shown in this part were ^13^C labeled. Different letters (a and b) mean significant differences between different treatments at the *P < *0.05 level.

Twenty-two COG pathways were predicted through 16S rDNA sequencing of ^13^C-labeled samples. These included all bacterial growth processes, such as reproduction, organic or inorganic nutrient metabolism, signaling, and immunity (Fig. 6). Eleven COG functional pathways, which contained over half of all the pathways, obtained significantly different abundance between *F. mosseae* and *G. margarita* in Exp1 (Fig. 6). In detail, the relative abundance of amino acid transport and metabolism, cell motility, coenzyme transport and metabolism, general function prediction only, intracellular trafficking, secretion, and vesicular transport and transcription was greater in the *G. margarita* hyphosphere microbiome, while the relative abundance of carbohydrate transport and metabolism, defense mechanisms, energy production and conversion, secondary metabolite biosynthesis, transport and catabolism and translation, ribosomal structure, and biogenesis were much more prevalent in the *F. mosseae* hyphosphere microbiome. Fifteen COG pathways showed a significant difference between *R. intraradices* and *G. margarita*, while eight of them were greater in the *R. intraradices* hyphosphere (Fig. 6). In detail, cell cycle control, cell division, chromosome partitioning, cell motility, coenzyme transport and metabolism, inorganic ion transport and metabolism, intracellular trafficking, secretion, and vesicular transport, posttranslational modification, protein turnover, chaperones, replication, recombination, and repair, and signal transduction mechanisms were much more prevalent in the *G. margarita* hyphosphere microbiome, while the relative abundance of carbohydrate transport and metabolism, cytoskeleton, defense mechanisms, lipid transport and metabolism, RNA processing and modification, secondary metabolite biosynthesis, transport and catabolism, and transcription were much greater in the *R. intraradices* hyphosphere microbiome. Most interestingly, carbohydrate transport and metabolism pathways represented over 6% of all the results, and *G. margarita* exhibited a smaller abundance of these pathways than *F. mosseae* and *R. intraradices* in Exp 1 and Exp 2, respectively (Fig. 6).

## DISCUSSION

### Validation of a novel method for separating out the impact of AM fungi on the soil microbiome.

Traditionally, mycorrhizal colonization is measured by staining and microscopic observation methods (25). In contrast, in this study we used qPCR to quantify the DNA copy number to indicate mycorrhizal fungus colonizing status with species-specific 18S rRNA primers. There is a background threshold of 100 copies in the AM fungus DNA qPCR process that dictates the presence or absence of AM fungi (19). Our results suggest that all inoculated treatments have many orders of magnitude more DNA copies than those of control treatments (Fig. 1a). In addition, no other nontargeted AM fungus was found in any sample through PCR using AM fungal species-specific primers. Such results suggest that all inoculated AM fungi were well colonized in the split-root system of cotton without contamination.

In this study, we compared the bacterial community that associated with the hyphae (representing the hyphosphere microbiome) with the bacterial community in the soil collected from HCs of nonmycorrhizal treatments (representing the bulk soil). As the diameter of AM fungal hyphae is so small that it is difficult to separate soil particles from the hyphae, we quantified the bacteria that were tightly colonizing on the hyphal surface to indicate the status of the hyphosphere bacterial community.

To avoid any influence of root exudates on the measurements, we set a 1-cm-wide buffer zone in which we added a sterilized mixture of glass beads and fine clay soil which was sieved through 30-μm nylon mesh. Our results showed that δ^13^C of the HC soils of the control treatments was the same as the background, suggesting no direct influence from root exudates on the microbial community in HCs. Therefore, all differences between hyphosphere and bulk soil or between the different AM fungal species can be attributed to the effects of hyphal exudation.

As the turnover rate of AM extraradical hyphae is fast (26), both vital and nonvital hyphae exist simultaneously; importantly, it is thought that the bacterial communities associated with these two types of hyphae may differ (20). To avoid these influences, we used a 7-day ^13^CO_2_ pulse-labeling approach in the last week before harvesting, which ensured that all the ^13^C-labeled extraradical mycelia were vital, because the potential turnover time of AM fungal hyphae is 5 to 6 days (26). We assume nonvital hyphae will not consume the ^13^C-labeled carbohydrates because the senescent hyphae form septa to cease protoplasm flow in hyphae. Therefore, the atom percentage of ^13^C of the samples in HCs indicated the allocation of photosynthetic products to vital extraradical hyphae and hyphae associated with soil particles and bacteria, and the ^13^C-DNA-SIP identified in the hyphosphere microbiome were active hypha exudate consumers.

### The influence of AM extraradical hyphal exudates on biophysical distribution of the soil microbial community and biodiversity.

Arbuscular mycorrhizal fungi produce a large network of extraradical hyphae in soil and provide a carbon-rich habitat for soil microbes (5, 6), which induces colonization of diverse groups of bacteria forming the hyphosphere (7, 27, 28). Our current study not only further supports those previous findings, but also provides novel findings. First, the differences in qPCR (Fig. 1a) and plant biomass (Table 1) results between *F. mosseae* and *G. margarita* and NM in Exp 1 or *R. intraradices* and *G. margarita* and NM in Exp 2 indicated that all AM fungal species colonized roots of cotton and played a role in promoting plant growth. Second, we successfully separated the active bacteria that consumed hyphal exudates by ^13^C-DNA-SIP plus MiSeq sequencing methods (Fig. S1). Compared to bulk soil, we found that only part of the soil microbiome was ^13^C-labeled on the hyphae of the AM fungi, which we defined as the active hyphosphere microbiome (Fig. S3). Third, the cocolonizing AM fungi all formed a unique bacterial community around the extraradical mycelium (Fig. S2 and S3). Our observations help us understand the biophysical mechanisms which dictate the heterogeneous distribution of the microbiome in soil at the microscale (29–31). Our current findings provide new and direct evidence that AM fungal hyphae, most likely through their exudates, are one of the major driving forces for formation of the bacteria mosaic at the micrometer scale in soil. As AM fungi use up to 20% of plant photosynthesis products and form several meters to tens of meters of hyphae in 1 g of soil (32), an understanding of such mechanisms has significance within the context of the global soil microbial biodiversity and its function.

10.1128/mSystems.00929-20.5 FIG S3 The principal-component analysis (PCA) of 16S rRNA genes from all 30 samples. The three different AM fungal inocula were *Rhizophagus intraradices* (*R.i*) (EY108), *Funneliformis mosseae* (*F.m*) (MD118), and *Gigaspora margarita* (*G.m*) (JA101A). All samples are shown in this part. Download FIG S3, TIF file, 0.1 MB. Copyright © 2020 Zhou et al. 2020 Zhou et al. This content is distributed under the terms of the Creative Commons Attribution 4.0 International license.

### Cocolonizing AM fungal species recruit different active hyphosphere microbial communities.

Previous studies have shown that a range of AM fungal species, which are different in morphological structure, hyphal distribution pattern, and metabolic traits, can simultaneously colonize a single root system (16, 33, 34). Whether or not these fungi recruit different microbiomes is still an open question.

We hypothesized that any difference in microbial community structures between the two HCs in Exp 1 and Exp 2 can be attributed to the differences in traits of excretion of exudates between the two AM fungal species. Our ^13^C-DNA-SIP plus pyrosequencing results supported the hypothesis. First, different AM fungal species produced differing amounts of hyphae in HCs (Fig. 1b). Compared to *G. margarita*, the HCs of both *F. mosseae* and *R. intraradices* contained a greater ^13^C abundance. Second, there were more OTUs in the microbiome of the *F. mosseae* and *R. intraradices* hyphosphere than in that of *G. margarita* in Exp 1 and Exp 2, respectively. More importantly, the abundance and structure of over half of the bacteria, at both the phylum and genus levels, showed a significant difference between *F. mosseae* and *G. margarita* in Exp 1 and between *R. intraradices* and *G. margarita* in Exp 2 (Fig. 2 to 5). All these results suggest that the microbiomes associated with the three AM fungal species were distinct.

Previous studies have indicated that the hyphosphere microbiome is directly involved in soil organic N, P, and C mineralization (7, 28, 32, 35, 36). For example, *Pseudomonas* and *Bacillus* are reported to have abilities to mobilize sparingly soluble P in soil (Table S1) (5, 7). In the current study, *G. margarita* harbored a greater abundance of *Pseudomonas*, but fewer *Bacillus*, than *F. mosseae* or *R. intraradices*. In addition, some soil bacteria, called mycorrhiza helper bacteria (MHB), can help AM fungi colonize the root more effectively or cause them to branch more (10). Such MHB belong to many taxa, including *Proteobacteria* (*Agrobacterium*, *Azospirillum*, *Azotobacter*, *Burkholderia*, *Bradyrhizobium*, *Enterobacter*, *Pseudomonas*, *Klebsiella*, and *Rhizobium*), *Firmicutes* (*Bacillus*, *Brevibacillus*, and *Paenibacillus*), *Actinomycetes* (*Rhodococcus*, *Streptomyces*, and *Arthrobacter*), and some unculturable bacterial taxa such as *Acidobacteria* (*Acidobacterium*) (37) (Table S2). However, MHB are often AM fungal species specific, which means they can stimulate mycorrhizal formation and extraradical hypha production for some AM fungi but inhibit these traits for the others (38). For example, *Streptomyces* spp. enhanced the colonization of *R. intraradices* (formerly named *Glomus intraradices*) but inhibited the growth of Hebeloma cylindrosporum (39, 40). Here, we found that the abundance of *Streptomyces* and *Bacillus* was much greater in the hyphosphere of *R. intraradices* and *F. mosseae* than in that of *G. margarita*, while *G. margarita* contained the largest abundance of *Pseudomonas*. These observations suggest that different AM fungal species might cooperate with different functional bacteria and have different impacts on the function of the hyphosphere. The COG functional prediction also supported this assertion, indicating that distinct microbiomes recruited by different AM fungi contained different abundances of inorganic P mobilization abilities or other functions (Fig. 6). Further studies are needed to investigate the functions of the hyphosphere microbiome in specific nutrition cycling.

10.1128/mSystems.00929-20.7 TABLE S1 The relative abundance (%) of phosphate-solubilizing bacteria (PSB) referred to in previous studies. Download Table S1, DOCX file, 0.01 MB. Copyright © 2020 Zhou et al. 2020 Zhou et al. This content is distributed under the terms of the Creative Commons Attribution 4.0 International license.

10.1128/mSystems.00929-20.8 TABLE S2 The relative abundance (%) of mycorrhizal helper bacteria (MHB) referred to in previous studies. Download Table S2, DOCX file, 0.02 MB. Copyright © 2020 Zhou et al. 2020 Zhou et al. This content is distributed under the terms of the Creative Commons Attribution 4.0 International license.

### Outlook and conclusion.

The soil microbiome is critical to the functioning of the plant-AM fungi-bacteria-soil particle continuum and therefore to growing food sustainably with minimal environmental impact and protecting against pathogens and disease while also providing important ecological services such as nutrient turnover and transformation and bioavailability. Understanding the structure of the microbiome is essential for using the native microbiome efficiently (41). In recent years, mycorrhizal genome sequencing studies have found that mycorrhizal fungi have lost many saprophytic genes in the long-term coevolution process with plants (17). Cooperating with functional microbiomes, such as phosphatase-releasing bacteria (6, 42), is considered an important strategy for AM fungi to compensate for their lack of ability to utilize organic P, for example. We find for the first time that different living AM fungus species colonizing a single plant root system recruit active microbiomes which are distinct from each other. The research not only provides direct evidence for understanding the biophysical process by which AM fungal hypha exudates drive the formation of soil bacteria diversity heterogeneity, but also reveals that the potential division of labor may exist in the plant-AM fungus-bacterium system that still remains to be understood fully. More knowledge of these key interactions in the hyphosphere has the potential to allow effective management of resources in agricultural systems and help us improve future agricultural sustainability.

## MATERIALS AND METHODS

### Soil.

A moderately acid soil (Inceptisol according to the USDA classification system) from Tai’an, Shandong Province, China (36°10′N, 117°09′E) was used. Physicochemical properties of the soil are as follows: pH (soil:H_2_O = 1:5), 6.5; organic matter, 5.8 g kg^−1^; mineral N, 7.2 mg kg^−1^; Olsen-P ([NaHCO_3_] extractable), 3.6 mg kg^−1^; NH_4_OAc exchangeable K, 37.6 mg kg^−1^. The collected soil was air dried and sieved (2 mm). The basal nutrients were added to the soil as described in Table S3. The soil was sterilized by gamma irradiation (25 kGy, 60 Co gamma rays) at the Beijing Atomic Energy Research Institute to eliminate indigenous microorganisms and mycorrhizal propagules before use. Previous studies have demonstrated that AM fungi recruit a hyphosphere microbiome that has the potential to stimulate the solubility of organic P (6, 7). In this study, to enhance the colonization of the soil microbiome in hyphosphere, 100 mg kg^−1^ myo-inositol hexaphosphate calcium magnesium salt (phytin; TCI, Tokyo, Japan) (equal to 20 mg P kg^−1^ soil) was added to the hyphal compartment as an organic P source. In order to induce the AM fungal hyphae to release protons to acidify the hyphosphere soil, (NH_4_)_2_SO_4_ was provided as the N source (43). In addition, a nitrification inhibitor 3,4-dimethylpyrazole phosphate (DMPP; ENTEC Flüssig produced by EuroChem Agro GmbH, Mannheim, Germany) was also added, at a rate of 1% (wt/wt) of the N applied to prevent nitrification of (NH_4_)_2_SO_4_.

10.1128/mSystems.00929-20.9 TABLE S3 Basal mineral nutrients added to the soil. Download Table S3, DOCX file, 0.01 MB. Copyright © 2020 Zhou et al. 2020 Zhou et al. This content is distributed under the terms of the Creative Commons Attribution 4.0 International license.

### Microcosms.

**FIG 7:**
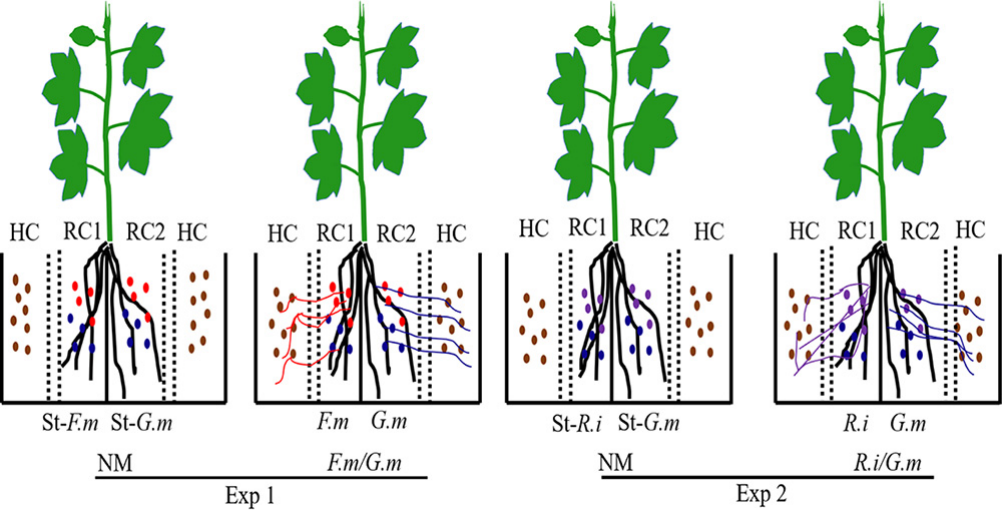
The experimental design and plant growth system. The host plant was cotton (*Gossypium herbaceum L.*). RC and HC denote the root compartment and hyphal compartment, respectively. The dotted lines indicate a 30-μm nylon mesh, and the zone between two meshes was a buffer zone. Exp 1 and Exp 2 refer to two independent experiments. The nonmycorrhizal (NM) control is compared to *Rhizophagus intraradices* (*R.i*) (EY108), *Funneliformis mosseae* (*F.m*) (MD118), and *Gigaspora margarita* (*G.m*) (JA101A), the three different AM fungal inocula. St means sterilized. The information on AM fungal inoculation treatments and inoculum filtrates supplied to RCs is shown in the Table S3. The brown circles represent the original bacterial community from soil. The red, blue, and purple lines represent the hyphae of *F.m*, *G.m*, and *R.i*, respectively, while the red, blue, and purple circles represent the original bacterial community from *F.m*, *G.m*, and *R.i* inoculum, respectively.

In order to test whether the extraradical mycelium of each AM fungal species that simultaneously colonized on the same root system would recruit their own microbiome, we used a split-root and compartmented microcosm system that separated the growing spaces of root systems and the extraradical mycelium of two AM fungal species, respectively (Fig. 7). The microcosms were constructed using polyvinyl chloride (PVC) plates and consisted of four compartments. The two middle compartments were separated by PVC plates and were used for split-root growth (root compartment, RCs). The two outer compartments (hyphal compartments, HCs) were separated from the RCs by a 1-cm buffer zone. The buffer zone consisted of two layers of 30-μm nylon mesh, which allowed AM fungal hyphae to pass through but prevented root penetration. In order to easily extract the hyphae, the fine soil used in the HCs was sieved with a 30-μm mesh. In short, fine soil was prepared by wet sieving. Approximately 1 kg of air-dried soil was placed in a 5-liter bucket, 3 to 4 liters of tap water was added, and the soil was brought into suspension by stirring. The soil suspension was poured through a sieve with a mesh width of 30 μm. This procedure was repeated three times on each 1-kg soil portion. The sieved soil suspension was collected in another bucket and allowed to settle until the water above the soil layer became clear and was siphoned off using a flexible tube. The remaining sludge was transferred to a shallow, heat-resistant dish and was dried at 60°C until the material became solid. The fine soil was then mixed with glass beads (1 mm in diameter) in a 1:1 (wt/wt) ratio. The mixture was sterilized by gamma irradiation (25 kGy, 60 Co gamma rays) at the Beijing Atomic Energy Research Institute. The microcosms received the following amounts of soil or soil-glass bead mixture: 500 g soil in each RC and 165 g soil-glass bead mixture in each buffer zone and 495 g soil-glass bead mixture in each HC. The soil or soil-glass bead mixture was filled very carefully into each compartment to maintain equal bulk density in each HC.

### Host plants.

Cotton (Gossypium herbaceum L., cv. Xinluzao 32) seeds were surface-sterilized with 10% (vol/vol) H_2_O_2_ (43) and germinated on moist filter paper for 2 days at 26°C in the dark. The seeds were then transferred to 40 × 25-cm moist filter paper for 17 days (12 h light, 12 h dark, 26°C) to allow the roots to grow longer. Seedlings of similar size and with nine roots (including taproot) were selected, and one plant was transplanted into each microcosm.

### AM fungal and bacterial inoculant.

The inoculums of *Rhizophagus intraradices* (EY108), *Funneliformis mosseae* (MD118) and *Gigaspora margarita* (JA101A) were purchased from the International Culture Collection of (Vesicular) Arbuscular Mycorrhizal Fungi (INVAM). They were propagated through hosts (maize: Nongda 108 and Plantago depressa Wild.) in zeolite and sand for 5 months; the spore density was about 20 spores g^−1^ substrate. In order to keep the same RC original microflora, 5 ml of AM fungal inoculum filtrate was added to each RC as described in Table S4. A total of 5 ml of soil filtrate was added to the hyphal compartment as the original hyphal compartment microflora. The filtrate of inoculum or soil was obtained by suspending 30 g of unsterilized inoculum or soil in 300 ml of sterile water and filtration through six-layer quantitative filter paper (properties similar to Whatman grade 43) (5), which allowed the passage of common soil microbes but effectively retained spores and hyphae of mycorrhizal fungi.

10.1128/mSystems.00929-20.10 TABLE S4 Arbuscular mycorrhizal inoculation treatments and inoculum filtrates supplied to RCs. Download Table S4, DOCX file, 0.01 MB. Copyright © 2020 Zhou et al. 2020 Zhou et al. This content is distributed under the terms of the Creative Commons Attribution 4.0 International license.

### Experimental design and procedure.

Two single-factor experiments were conducted, experiment 1 (Exp 1) and experiment 2 (Exp 2), which are described in Fig. 7. There were 6 replicates for each treatment; 3 were labeled with ^13^CO_2_, while the other 3 were given ^12^CO_2_ treatment as a control. At planting, half of the soil for each RC (250 g) was carefully added to the RC, and then 30 g AM fungal inoculum of each AM fungal species containing about 600 spores was added to each RC. The taproot of the precultured plant was cut off at the elongation zone, and the shoot was mounted on the central PVC plate. The two groups of lateral roots were evenly separated into the two RCs. Finally, the remaining 250 g soil was added to the RCs. The control treatments (NM) in both experiments received the same amount of sterilized inoculum. The HCs and buffer zone were filled with a soil-glass bead mixture, and thus, the substrates in the four sections are referred to as root soil, buffer soil, and hyphal soil (Fig. 7). Plants in these microcosms were grown in a greenhouse at China Agricultural University in Beijing, China, from 13 May to 8 July 2015 at 24/30°C (night/day) and an average photosynthetically active radiation of 360 μmol m^−2^ s^−1^. To avoid any possible influence of environmental factors in the glasshouse, the position of the microcosms was rerandomized once a week. Soil gravimetrical moisture was kept at 18 to 20% (wt/wt, ∼70% water-holding capacity) with deionized water added to the weight every 2 days during the experiment.

### ^13^CO_2_ pulse-labeling chamber and procedure.

To trace the transfer of plant-derived C from mycorrhizal hyphae to the hyphosphere microbes, ^13^CO_2_ stable isotope pulse-labeling was conducted in the glasshouse for 7 days before harvest. Then, 7 weeks after sowing, the cotton plants were subjected to ^13^CO_2_ (99% of ^13^C atom) pulse-labeling in an airtight Plexiglas growth chamber (Fig. S4). The plant shoots protruded through the holes, and the joins between stems and chamber were sealed with silica gel to prevent direct exposure of the soil surface to the ^13^CO_2_ labeling. During pulse-labeling, a cooling system was used to cool the chamber temperature to 35°C. A 100-ml aliquot of ^13^CO_2_ was injected through the septum using a gas-tight syringe every hour from 9 a.m. to 5 p.m., the period in which photosynthesis was the greatest during the day (44). During this process, the CO_2_ concentration was measured using an infrared gas analyzer. The CO_2_ concentration reached about 450 μM after injection and about 10 μM before injection. The lid was removed 1 h after the last CO_2_ injection, when the ^13^CO_2_ concentration in the chamber had decreased to atmospheric levels. The plants were labeled for 7 days. Simultaneously, the same procedures of ^12^CO_2_ (99% of ^12^C atom) labeling control were also performed (5). To remove the influence of vapor produced by plant evaporation during CO_2_ labeling on photosynthesis, three trays of CaCl_2_ (100 g per tray) were placed in the chamber. The wet CaCl_2_ trays were removed and dried in a forced-air oven at 105°C for 2 h every day after the lid of the chamber was removed in the evening and were reused repeatedly.

10.1128/mSystems.00929-20.6 FIG S4 Root growth system and ^13^CO_2_ isotope probing equipment. The growing system was made up of a combination of four compartments, as detailed in Fig. 7. Cotton roots were divided equally into two root compartments by the PVC plate to prevent the interaction between divided roots. The probing equipment included a holder, a cover, and a cooling system. The holder was 30 cm in height and consisted of a hyaline acrylic plate. A 5-cm-height water channel and a 5-cm-diameter hole were in the top of the holder. The cover also used a hyaline acrylic plate, which was sealed with a holder by water in a water channel. The cooling system consisted of a semiconductor, air fan, radiator, pump, heat sink, and two water pipes. Water was used to cool the setup by exchanging heat through the heat sink. When probing, plant shoots were passed through the hole and sealed with Vaseline to separate the atmosphere from the soil. Download FIG S4, TIF file, 0.3 MB. Copyright © 2020 Zhou et al. 2020 Zhou et al. This content is distributed under the terms of the Creative Commons Attribution 4.0 International license.

### Harvest and sample analysis.

The plants were harvested 8 weeks after planting. To prevent contamination of the hyphal samples with exotic bacteria settling on the surface soil, we removed the top 1 cm of soil to reduce any potential contamination. The soil in the buffer zone was removed before collecting the soil from the hyphal compartment. Soil from two HCs of NM treatments was mixed as one sample. A part of the soil was stored at 4°C for soil analysis, and another part was immediately frozen in liquid nitrogen and stored at –80°C until DNA extraction for microbial diversity tests could be performed. The shoots were oven-dried before measuring the dry weight and processing for shoot P concentration. Determination of shoot P concentration was performed according to the method of Thomas et al. (45).

Root DNA was extracted using a plant genome kit (Tiangen Co. Ltd., Beijing, China) following the manufacturer’s instructions, and the AM fungal gene copies were detected to assess the AM fungal root colonization rate. The AM fungi copies were quantified in triplicate with real-time qPCR using a q-TOWER qPCR analyzer (Jena, Germany) with root DNA extracted from each treatment with AM fungus-specific primers (Table S5) and using the methods described in the supplemental material. The hyphal length density of HC soil was determined according to the method of Jakobsen et al. (46).

10.1128/mSystems.00929-20.11 TABLE S5 Details of primers used in this experiment. Download Table S5, DOCX file, 0.02 MB. Copyright © 2020 Zhou et al. 2020 Zhou et al. This content is distributed under the terms of the Creative Commons Attribution 4.0 International license.

### ^13^C DNA stable isotope probing (SIP) analysis.

Soil samples stored at 4°C were oven-dried at 70°C, ground, sieved using an 80-μm mesh, and then the δ^13^C‰ was determined at the Stable Isotope Laboratory of the College of Resources and Environmental Sciences, China Agricultural University, Beijing, China (see details in the supplemental material). These soils were assumed to only contain ^13^C contained in AM fungal extraradical hyphae or released by the hyphae to the soil.

### Collection of extraradical mycelia from the hyphal compartment.

A total of 500 g of the soil, glass beads, and associated fungal material in the hyphal compartments was transferred to a sieve with a 30-μm mesh. The soil was carefully washed through the mesh with filtered sterile deionized water, leaving the extraradical mycelia and glass beads on the sieve. To separate the extraradical mycelia from the glass beads and to clean them, the mixture was transferred into a 1-liter beaker, and filtered sterile deionized water was added, and then the mixture was stirred and poured back into the sieve, leaving the glass beads in the beaker. This procedure was repeated five times. The extraradical mycelia were rinsed with filtered sterile deionized water before they were collected from the sieve using forceps and placed into a microcentrifuge tube. All mycelia samples were weighed before DNA extraction and afterward were stored at −80°C until further processing.

For the nonmycorrhizal treatments, no extraradical hyphae were observed in the hyphal compartment when samples were collected as described above. A subsample of 0.5 g residual soil particles on the sieve was collected and referred to as nonmycorrhizal samples. These samples were also stored at −80°C before DNA extraction and tagged as NM (nonmycorrhizal treatment).

### DNA extraction, density gradient centrifugation, and qPCR analysis.

DNA of AM fungal mycelia and soil samples collected from the last step was extracted using the FastDNA SPIN kit (MP Biomedicals LLC, Santa Ana, CA, USA) following the manufacturer’s instructions. All the extracted rDNA samples (approximately 500 ng) were fully blended with cesium trifluoroacetate (CsTFA) to achieve an initial buoyant density (BD) of 1.560 g ml^−1^ before ultracentrifugation at 45,400 rpm for 36 h (47). The centrifuged gradients were fractionated from bottom to top into 16 equal fractions. The buoyant density of DNA in the gradient fractions was determined using a digital refractometer (Reichert AR2000). The DNA fractions were then purified with isopropyl alcohol and 70% (vol/vol) ethanol and stored at −80°C for further analysis. DNA from each gradient fraction of all treatments was quantified in triplicate with real-time qPCR using a q-TOWER qPCR analyzer (Jena, Germany) with primers Ba519f/Ba907r (Table S5) using the protocol described in the supplemental material.

### 16S rRNA gene-based MiSeq sequencing.

Fractions which had a buoyant density of approximately 1.58 were quality checked, and then the DNA samples were sent to the Majorbio Biotechnology Company (Shanghai, China) for sequencing on an Illumina MiSeq (PE300) sequencing platform. The V3-V4 hypervariable regions of 16S rDNA were amplified using primer set Ba338f/Ba806r (Table S5). The DNA samples from the NM control soil were sent for sequencing and used as the original soil microbial community. The DNA samples of AM fungal mycelia were considered the hyphosphere microbiome. In addition, ^12^C-labeled samples were sequenced and used as the whole hyphosphere microbiome, while ^13^C-labeled samples were used as the active hyphosphere microbiome, which was influenced by hyphal exudates directly.

### Processing of sequencing data.

The Quantitative Insights into Microbial Ecology (QIIME) v1.8.0 pipeline was used to process the sequencing data, as described previously (48). The raw sequencing reads were identified to operational taxonomic units (OTUs) according to the methods described in the supplemental material.

### Statistical analysis.

Split-root experiment data from the two experiments were analyzed separately. Data from *F. mosseae* and *G. margarita* HCs in Exp 1 or *R. intraradices* and *G. margarita* HC in Exp 2 were compared to determine the difference between different AM fungal species. Data from the nonmycorrhizal (NM) control were also compared with *F. mosseae* and *G. margarita* in Exp 1 or *R. intraradices* and *G. margarita* in Exp 2 to determine the effect of AM fungal inoculation. Before analysis of variance (ANOVA), the AM fungal DNA copy number was used to assess the mycorrhizal colonization rate and was log-10 transformed. Likewise, the data for the relative abundance of ^13^C in HC soil, Simpson diversity index, taxon groups (phyla and genera), and Cluster of Ortholog Genes (COG) functional pathways were arcsine-transformed. SPSS 21.0 (Statistical Product and Service Solutions; IBM, USA) was employed to conduct the above-described analysis.

Shoot biomass, P concentration, and content data were analyzed separately for the NM or AM (*F. mosseae*/*G. margarita* or *R. intraradices*/*G. margarita*) as the treatment factor. *A posteriori* comparison was made using Tukey tests (*P* < 0.05) with SPSS 16.0. SPSS 21.0 (Statistical Product and Service Solutions; IBM, USA) was employed to conduct the above-described analysis.

The rarefaction curve of OTUs for each treatment was calculated with Usearch 7.0. The sequencing results of the ^13^CO_2_ pulse-labeling samples were used to stand for the active hyphosphere microbial community.

Bray-Curtis distances of 16S rRNA genes in nonmetric principal-component analysis (PCA) were calculated with QIIME software and then analyzed with the vegan package in R 2.4.2 to compare the β-diversity of each experiment. The significance of the data was estimated using Adonis with *P* < 0.05 with the vegan package in R 2.4.2.

The OTUs of 16S rDNA were standardized using PICRUSt (PICRUSt software stores COG information corresponding to the Greengene ID), and the COG family information corresponding to each OTU through the Greengene IDcorresponding to each OTU for functional prediction was obtained.

### Data availability.

The sequences obtained in this study were deposited in the GenBank database under accession number PRJNA556534. The Illumina MiSeq sequence data sets are available at the NCBI Sequence Read Archive BioProject number PRJN556534.

10.1128/mSystems.00929-20.1 TEXT S1 Real-time qPCR analysis protocol; ^13^C DNA stable isotope probing (SIP) analysis; processing of pyrosequencing data. Download Text S1, DOCX file, 0.02 MB. Copyright © 2020 Zhou et al. 2020 Zhou et al. This content is distributed under the terms of the Creative Commons Attribution 4.0 International license.
