# Supplementary figures and images for: Different Arbuscular Mycorrhizal Fungi Cocolonizing on a Single Plant Root System Recruit Distinct Microbiomes
Source: mSystems. 2020 Dec 15;5(6):e00929-20. doi: 10.1128/mSystems.00929-20 (PMC7771537; doi:10.1128/mSystems.00929-20)

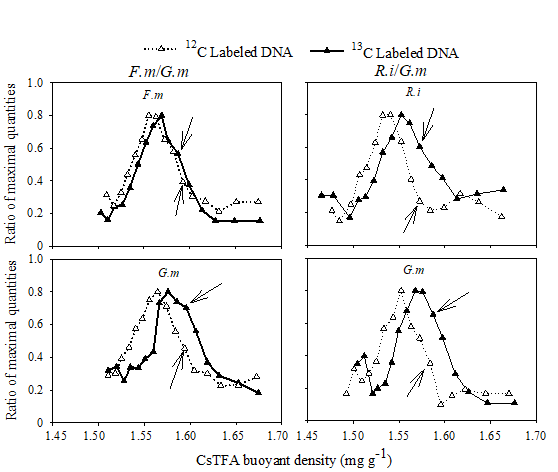

Supplement: FIG S1 [file mSystems.00929-20-sf001.tif]

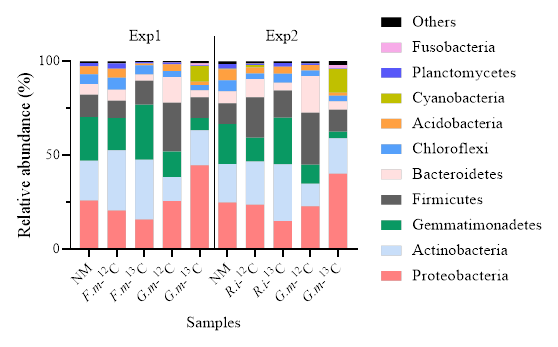

Supplement: FIG S2 [file mSystems.00929-20-sf002.tif]

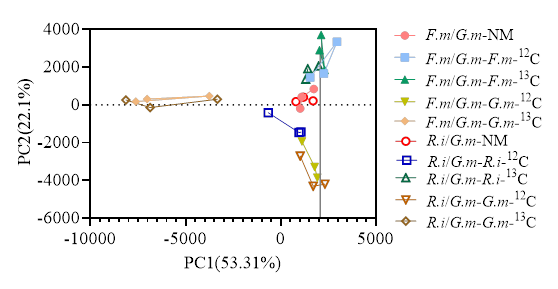

Supplement: FIG S3 [file mSystems.00929-20-sf003.tif]

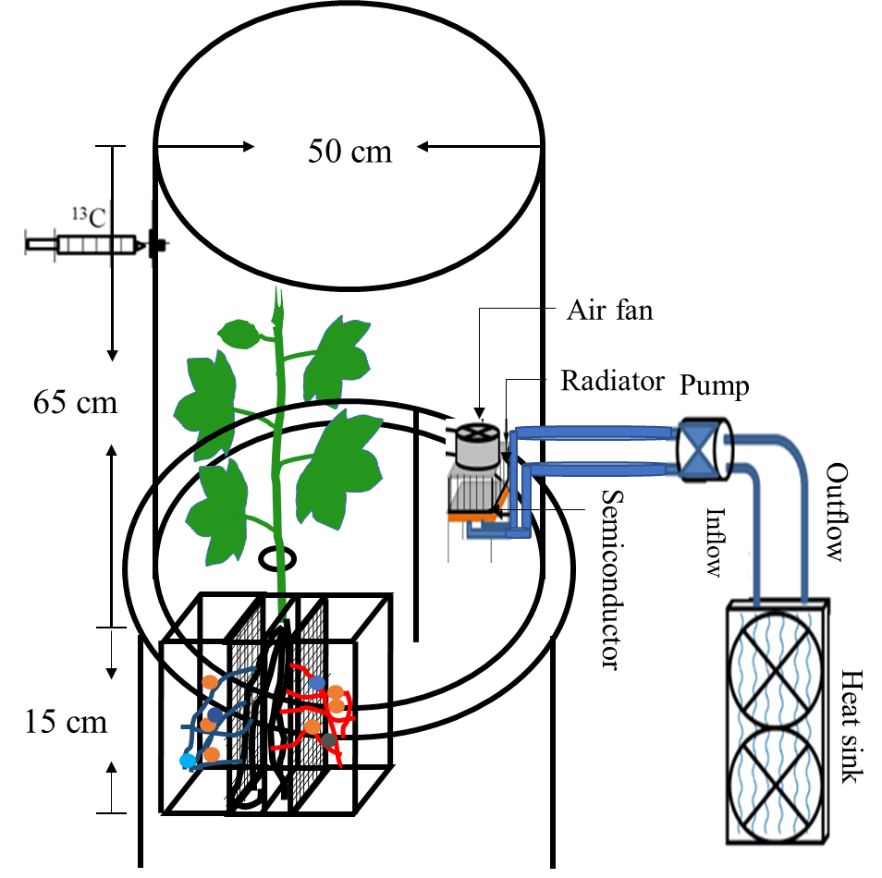

Supplement: FIG S4 [file mSystems.00929-20-sf004.tif]
