# Supplementary material for: Different Arbuscular Mycorrhizal Fungi Cocolonizing on a Single Plant Root System Recruit Distinct Microbiomes
Source: mSystems. 2020 Dec 15;5(6):e00929-20. doi: 10.1128/mSystems.00929-20 (PMC7771537; doi:10.1128/mSystems.00929-20)
Supplement: TABLE S1 [file mSystems.00929-20-st001.docx]

**Table S1.** The relative abundance (%) of phosphate solubilizing bacteria (PSB) referred to previous studies.

| PSB | | Exp1 | | Exp2 | |
| --- | --- | --- | --- | --- | --- |
|  | |  | |  | |
| Phylum | Genus | *F.m* | *G.m* | *R.i* | *G.m* |
| Proteobacteria | *Pseudomonas* | 0.27 | 21.37 | 0.24 | 18.0 |
|  | *Rhizobium* | 0.01 | 0.12 | 0.01 | 0.13 |
|  | *Burkholderia* | 0.02 | 0.00 | 0.02 | 0.00 |
|  | *Achromobacter* | 0.04 | 0.36 | 0.10 | 0.23 |
| Actinobacteria | *Agrobacterium* | 0.00 | 0.00 | 0.05 | 0.00 |
|  | *Microccocus* | 0.00 | 0.03 | 0.03 | 0.16 |
| Firmicutes | *Bacillus* | 7.38 | 2.71 | 8.49 | 2.16 |
| Bacteroidetes | *Flavobacterium* | 0.00 | 0.03 | 0.00 | 0.06 |
