# Supplementary material for: Different Arbuscular Mycorrhizal Fungi Cocolonizing on a Single Plant Root System Recruit Distinct Microbiomes
Source: mSystems. 2020 Dec 15;5(6):e00929-20. doi: 10.1128/mSystems.00929-20 (PMC7771537; doi:10.1128/mSystems.00929-20)
Supplement: TABLE S2 [file mSystems.00929-20-st002.docx]

**Table S2.** The relative abundance (%) of mycorrhizal helper bacteria (MHB) referred to previous studies.

| MHB | | Exp1 | | Exp2 | |
| --- | --- | --- | --- | --- | --- |
| Phylum | Genus | *F.m* | *G.m* | *R.i* | *G.m* |
| Proteobacteria | *Pseudomonas* | 0.27 | 21.37 | 0.24 | 18.0 |
|  | *Azospirillum* | 0.21 | <0.01 | 0.13 | 0.02 |
|  | *Klebsiella* | <0.01 | 0.10 | <0.01 | 0.10 |
|  | *Rhizobium* | <0.01 | 0.12 | <0.01 | 0.13 |
|  | *Enterobacter* | <0.01 | 0.10 | <0.01 | 0.04 |
|  | *Bradyrrhizobium* | 0.11 | 0.19 | 0.16 | 0.39 |
| Actinobacteria | *Agrobacterium* | <0.01 | <0.01 | <0.01 | 0.05 |
|  | *Streptomyces* | 7.00 | 0.63 | 5.97 | 0.45 |
| Firmicutes | *Bacillus* | 7.38 | 2.71 | 8.49 | 2.16 |
|  | *Paenibacillus* | 1.03 | 0.46 | 1.12 | 0.22 |
|  | *Brevibacillus* | 1.33 | 0.31 | 1.31 | 0.14 |
