# Supplementary material for: Different Arbuscular Mycorrhizal Fungi Cocolonizing on a Single Plant Root System Recruit Distinct Microbiomes
Source: mSystems. 2020 Dec 15;5(6):e00929-20. doi: 10.1128/mSystems.00929-20 (PMC7771537; doi:10.1128/mSystems.00929-20)
Supplement: TABLE S3 [file mSystems.00929-20-st003.docx]

**Table S3.** Basal mineral nutrients added to the soil.

| Nutrition | Nutrient form | Addition (mg kg^-1^) |
| --- | --- | --- |
| N | (NH4)_2_SO_4_ | 200 |
| K | K_2_SO_4_ | 200 |
| Mg | MgSO_4_·7H_2_O | 50 |
| Zn | ZnSO_4_·7H_2_O | 5 |
| Mn | MnSO_4_·H_2_O | 5 |
| Cu | CuSO_4_·5H_2_O | 2 |
| P | KH_2_PO_4_ | 30 |
