# Supplementary material for: Different Arbuscular Mycorrhizal Fungi Cocolonizing on a Single Plant Root System Recruit Distinct Microbiomes
Source: mSystems. 2020 Dec 15;5(6):e00929-20. doi: 10.1128/mSystems.00929-20 (PMC7771537; doi:10.1128/mSystems.00929-20)
Supplement: TABLE S4 [file mSystems.00929-20-st004.docx]

| Experiments | | Treatments | RC1 | RC2 |
| --- | --- | --- | --- | --- |
| Exp 1 | NM | | Sterilized *F. mosseae* + filtrates of mixed inoculum of *F. mosseae* and *G. margarita* | Sterilized *G. margarita* + filtrates of mixed inoculum of *F. mosseae* and *G. margarita* |
|  | *F.m*/*G.m* | | *F. mosseae* + filtrates of inoculum of *G. margarita* | *G. margarita* + filtrates of inoculum of *F. mosseae* |
| Exp 2 | NM | | Sterilized *R. intraradices* + filtrates of mixed inoculum of *R. intraradices* and *G. margarita* | Sterilized *G. margarita* + filtrates of mixed inoculum of *R. intraradices* and *G. margarita* |
|  | *R.i*/*G.m* | | *R. intraradices* + filtrates of inoculum of *G. margarita* | *G. margarita* + filtrates of inoculum of *R. intraradices* |

**Table S4** Arbuscular mycorrhizal inoculation treatments and inoculum filtrates supplied to RCs*.*
