# Supplementary material for: Different Arbuscular Mycorrhizal Fungi Cocolonizing on a Single Plant Root System Recruit Distinct Microbiomes
Source: mSystems. 2020 Dec 15;5(6):e00929-20. doi: 10.1128/mSystems.00929-20 (PMC7771537; doi:10.1128/mSystems.00929-20)
Supplement: TABLE S5 [file mSystems.00929-20-st005.docx]

**Table S5.** Details of primers used in this experiment.

| Gene | Name of primer | Sequence of primer | reference |
| --- | --- | --- | --- |
| 16S rDNA | Ba338f | ACTCCTACGGGAGGCAGCAG | (53) |
|  | Ba806r | GGACTACHVGGGTWTCTAAT |  |
| 16S rDNA | Ba519f | CAGCMGCCGCGGTAANWC | (54) |
|  | Ba907r | CCGTCAATTCMTTTRAGTT |  |
| AM fungi | AMV4.5NF | AAGCTCGTAGTTGAATTTCG | (55) |
|  | AMDGR | CCCAACTATCCCTATTAATCAT |  |

**Reference**

53. Xu N, Tan G, Wang H, Gai XP. 2016. Effect of biochar additions to soil on nitrogen leaching, microbial biomass and bacterial community structure. Eur J Soil Biol 74:1-8. https://doi.org/ 10.1016/j.ejsobi.2016.02.004.

54. Stubner S. 2002. Enumeration of 16S rDNA of Desulfotomaculum lineage 1 in rice field soil by real-time PCR with SybrGreen detection. J Microbiol Methods 50:155-164. https://doi.org/ 10.1016/S0167-7012(02)00024-6.

55. Sato K, Suyama Y, Saito M, Sugawara K. 2005. A new primer for discrimination of arbuscular mycorrhizal fungi with polymerase chain reaction-denature gradient gel electrophoresis. Grassl Sci 51:179-181. https://doi.org/ 10.1111/j.1744-697X.2005.00023.x.
