# Supplementary material for: Different Arbuscular Mycorrhizal Fungi Cocolonizing on a Single Plant Root System Recruit Distinct Microbiomes
Source: mSystems. 2020 Dec 15;5(6):e00929-20. doi: 10.1128/mSystems.00929-20 (PMC7771537; doi:10.1128/mSystems.00929-20)
Supplement: TEXT S1 [file mSystems.00929-20-sd001.docx]

**Supplementary 1**

**Real time q-PCR analysis protocol**

SYBR Green real time q-PCR Master Mix（TOYOBO, Japan）mix was used and conducted under the following reaction conditions: 5 min of initial denaturation at 94°C; 40 cycles of 30 s of denaturation at 94°C, 45 s of annealing at 60°C, and 1 min of elongation at 72°C. Fluorescence of SYBR green was detected after every cycle. The dissolution curve was collected when the whole reaction ended in 0.5ºC increments from 65ºC to 95ºC. The amplification of the PCR reactions had an efficiency of 1.99, where 2 is the highest quality representing doubling each amplification cycle (49) and an error value of 0.002 calculated as the mean squared error of the standard curve. No amplification was detected in the negative controls.

**^13^C DNA stable isotope probing (SIP) analysis**

The carbon isotope ratios of soil samples were determined using a DeltaPlusXP mass spectrometer (Thermo Scientifc, Bremen, Germany) coupled with an elemental analyzer (FlashEA 1112; CE Instruments, Wigan, UK) in the continuous ﬂow mode at the Stable Isotope Laboratory of the College of Resources and Environmental Sciences, China Agricultural University, Beijing, China. The elemental analyzer combustion temperature was 1020ºC. The carbon isotopic ratios were reported in the delta notation relative to the V-PDB (Vienna-Pee Dee Belemnite) standard using the following equation according to Deniro and Epstein (1978) (50):

δ^13^C=-(R_sample_/R_PDB_)×1000

Where δ^13^C is the carbon isotope ratio of the sample in parts per thousand (‰), and R_sample_ and R_PDB_ are the ^13^C/^12^C ratios of the sample and standard, respectively. The SD for the δ^13^C measurements was <0.15‰.

**Processing of pyrosequencing data**

The Quantitative Insights Into Microbial Ecology (QIIME, v1.8.0) pipeline was employed to process the sequencing data. Brieﬂy, raw sequencing reads with exact matches to the barcodes were assigned to respective samples and identiﬁed as valid sequences. The low-quality sequences were filtered using the following criteria: sequences that had a length of <150 bp, sequences that had average Phred scores of <20, sequences that contained ambiguous bases, and sequences that contained mononucleotide repeats of >8 bp. Paired-end reads were assembled using FLASH. After chimera detection, the remaining high-quality sequences were clustered into operational taxonomic units (OTUs) at 97% sequence identity by UCLUST (51). A representative sequence was selected from each OTUs using default parameters. Operational taxonomic unit classification was conducted by BLAST searching the representative sequences set against the Greengenes Database (52) using the best hit. An OTU table was further generated to record the abundance of each OTUs in each sample and the taxonomy of these OTUs. Any OTUs containing less than 0.001% of total sequences across all samples were discarded. To minimize the difference of sequencing depth across samples, an average, rounded rarefied OTUs table was generated by averaging 100 evenly resampled OTUs subsets under the 90% of the minimum sequencing depth for further analysis. Initial classification of sequences was performed in QIIME using the NCBI database (https://www.ncbi.nlm.nih.gov/) to obtain taxonomy information.

**Reference**

49. Tellmann G. 2008. The E-Method: a highly accurate technique for gene-expression analysis. Nat Methods 3:1–2. https://doi.org/10.1038/nmeth894.

50. Deniro MJ, Epstein S. 1978. Carbon Isotopic Evidence for Different Feeding Patterns in Two Hyrax Species Occupying the Same Habitat. Science 201:906-908. https://doi.org/10.1126/science.201.4359.906.

51. Edgar RC. 2010. Search and clustering orders of magnitude faster than BLAST. Bioinformatics 26:2460-2461. https://doi.org/ 10.1093/bioinformatics/btq461.

52. DeSantis TZ, Hugenholtz P, Larsen N, Rojas M, Brodie EL, Keller K, Huber T, Dalevi D, Hu P, Andersen GL. 2006. Greengenes, a chimera-checked 16S rRNA gene database and workbench compatible with ARB. Appl Environ Microbiol 2:5069-5072. https://doi.org/ 10.1128/AEM.03006-05.
